# Tuberculous Disease of the Knee-Joint

**Published:** 1908-10-17

**Authors:** 


### Surgery.

### TUBERCULOUS DISEASE OF THE KNEE-JOINT.

### III.—OPERATIVE TREATMENT.

If, in spite of palliative treatment patiently persisted in, the local condition of the joint steadily progresses from bad to worse, it will finally become necessary to have recourse to an operation. The chief indications for this are the presence of abscesses and sinuses, and the appearance of the characteristic deformities already mentioned, if they cannot be overcome by extension. But even when an abscess has formed it may be possible to arrest the disease by making an incision into it and draining it, provided the patient can be kept in proper hygienic surroundings. Of these, the most important is fresh air, an item which is just as essential in surgical tuberculosis as it is in the pulmonary variety. In proof of which, it is less often found necessary to proceed to extreme surgical measures in the treatment of this disease at Margate, where the air is said to have special curative properties, than it is in London.

The surgeon has the choice of two operations: (*a*) arthrectomy, or erasion of the joint, and (*b*) excision. This will depend largely upon the state of affairs which is found when the joint is opened. The former operation consists in opening the joint, removing the diseased synovial membrane, and scraping with a sharp spoon a thin layer from the articular surfaces. It stands to reason therefore that this operation is of avail only when the synovial membrane, and that alone, is affected. But in the advanced stages which call for operation there is nearly always caries of one or other articular end, and if any operation is called for at all, a more radical one is needed.

Excision of the knee-joint is performed as follows: A large semilunar incision is made which starts above one condyle, passes across the leg at the level of the tubercle of the tibia, and is then carried up to a corresponding point on the opposite side. The ligamentum patellæ is divided, and the flap, so formed, is turned up, exposing the cavity of the joint. The structures which unite the femur to the tibia are divided, and the ends of the bones exposed by flexing the leg at the knee acutely. The lower end of the femur is sawn off first. The bone is firmly held in a vertical position by the assistant while the surgeon saws through the bone. Care must be taken that the epiphysial line is not encroached upon, or subsequent growth will be inhibited in a young patient, and this will lead to great shortening, since growth in the leg takes place mainly in the lower end of the femur, and the upper end of the tibia. It is also of the utmost importance to see that the plane of the sawn surface is at right angles to the shaft of the bone. It is well not to saw through the bone altogether, but to finish the removal of the lower end, levering the saw sideways when the section is almost complete. This precaution is necessary on account of the propinquity of the popliteal artery, which may be damaged if the saw is carried right through. A peculiar change in the shape of the lower end of the femur is sometimes observed in this disease, namely a deepening of the intercondyloid notch. It is due to the rarefaction of the bone by the tuberculous disease, and is of no practical importance. The upper end of the tibia is next dealt with in an exactly identical manner. When the ends of the bones have been removed attention must be directed to clearing out any outlying foci of disease, either in the exposed ends or in the crevices of the synovial membrane. There is some difference of opinion among surgeons as to whether the patella should be removed, or left *in situ*. There can be no question that if the disease has involved its articular surface it should be removed entirely; and in any case it serves no purpose in the ankylosed limb, and it is therefore better to do this in all cases as a routine measure. The sawn surfaces are then brought into contact by extending the limb, and any bleeding points which can be seen are ligatured, and the wound sewn up. It is well to put a small drainage tube in one angle of the wound, since a large cavity is left into which a good deal of oozing takes place. This can be removed in forty-eight hours.

During the after-treatment the limb is treated in the same manner as a compound fracture, which indeed it is; that is to say, it is put on a back splint with side splints until union between the ends of the bones takes place. The greatest difficulty which confronts the surgeon at this stage of the case is that of preventing flexion from occurring, so that an angle is produced between the two bones. This, when it happens, is due to the same cause as produces the triple displacement previously mentioned, *i.e.* to the unopposed action of the biceps femoris. It has therefore been suggested that it would be a good plan to unite the ends of the bones mechanically at the time of operation, either by means of silver wire or a metal screw, and some surgeons make a habit of doing this. But the system often fails to prevent the deformity, and it has a further grave disadvantage, that the wire or screw acts as a chronic irritation, which damages the vitality of the bone so that a fresh focus of tuberculosis may make its appearance round the foreign body. It would seem more reasonable to attempt to overcome the power of the biceps muscle by dividing it at the time of operation.

The whole object of the operation is to remove the disease, and obtain union of the femur and tibia in one straight line, and if this is achieved the patient is left with a limb which is necessarily shorter than the sound one, but one on which he can not only support himself in comfort, but can also walk with moderate ease, if he is supplied with a proper mechanical appliance. But even though the result is as successful as is humanly possible, the final condition is not one of which the surgeon has reason to feel proud, because it is not to be compared with successful palliative treatment.

